# Supplementary material for: Intake of MPRO3 over 4 Weeks Reduces Glucose Levels and Improves Gastrointestinal Health and Metabolism
Source: Microorganisms. 2021 Dec 31;10(1):88. doi: 10.3390/microorganisms10010088 (PMC8780283; doi:10.3390/microorganisms10010088)
Supplement: Supplementary file 1 [file microorganisms-10-00088-s001.zip › Table S1.pdf]

Table S1. Ingredients of Mpro3 and Placebo.

Ingredients	MPRO3		Placebo	
Single dose/one-day intake	1 bottle (260 mg of capsule (130 mg X 2 capsules), 130 ml of liquid)			
Calorific value	130	kcal	120	kcal
Carbohydrate	25g	(8%)	19g	(6%)
Sugar content	12g	(12%)	18g	
Protein	3g	(5%)	2g	(4%)
Fat	4g	(7%)	4g	(7%)
Sodium	40mg		20mg	(1%)
Fiber	8g	(32%)		
xylo-oligosaccharide	700mg			
Probiotic	10,000,000,000CFU			

*() the figure in the proposal is the ratio of the daily nutritional standard
