# Supplementary material for: Intake of MPRO3 over 4 Weeks Reduces Glucose Levels and Improves Gastrointestinal Health and Metabolism
Source: Microorganisms. 2021 Dec 31;10(1):88. doi: 10.3390/microorganisms10010088 (PMC8780283; doi:10.3390/microorganisms10010088)
Supplement: Supplementary file 1 [file microorganisms-10-00088-s001.zip › Table S3.pdf]

Table S3. Characteristics and nutrient intake profile in the subjects before intervention.

	A (n= 17)		B (n= 18)		C (n= 16)		***P***	***P´***
Age (years)	71.35	±0.89	70.28	±1.03	68.69	±1.26	0.223	
Body mass Index (kg/m^2)	23.81	±0.77	23.39	±0.66	24.15	±0.81	0.773	
Energy intake (kcal)	1,721.65	±113.35[b]	1,695.57	±94.83[b]	1,343.64	±82.46[a]	0.017*	
Carbohydrate (g)	238.38	±17.46[b]	254.29	±17.54[b]	174.76	±12.29[a]	0.003**	0.092
Protein (g)	79.21	±6.36[b]	64.94	±5.97[ab]	57.25	±4.63[a]	0.033*	0.025*
Fat (g)	50.22	±6.26	47.53	±16.05	46.67	±5.85	0.888	0.146
Dietary fiber (g)	28.54	±3.04	28.20	±20.61	24.70	±2.75	0.578	0.371

Values are Mean±S.E.

Values with different letters([a-b]) within the same column differ significantly($p<0.05$) through one-way ANOVA followed by Duncan`s multiple range test.

$P^{*}<0.05$, $P^{**}<0.01$

P´: adjusted for energy intake
