# Supplementary material for: Intake of MPRO3 over 4 Weeks Reduces Glucose Levels and Improves Gastrointestinal Health and Metabolism
Source: Microorganisms. 2021 Dec 31;10(1):88. doi: 10.3390/microorganisms10010088 (PMC8780283; doi:10.3390/microorganisms10010088)
Supplement: Supplementary file 1 [file microorganisms-10-00088-s001.zip › Table S4.pdf]

Table S4. Characteristics and nutrient intake profile in the subjects before, during, and after intervention.

	A (n= 17)			B (n= 18)			C (n= 16)			P_1	P_2	P_3
	0 wk	1 wk	4 wk	0 wk	1 wk	4 wk	0 wk	1 wk	4 wk			
Body mass Index (kg/m^2)	23.81 ±0.77	23.96 ±0.75	23.85 ±0.75	23.39 ±0.66	23.40 ±0.67	24.23 ±1.01	24.15 ±0.81	24.10 ±0.79	23.94 ±0.85	0.773	0.772	0.949
Energy intake (kcal)	1,721.65 ±113.35^{b}	1,758.60 ±128.0	1,673.06 ±133.82	1,695.57 ±94.83^{b}	1,690.93 ±98.24	1,673.07 ±120.27	1,343.64 ±82.46^{a}	1,523.07 ±105.52	1,517.16 ±118.77	0.017*	0.326	0.607
Carbohydrate (g)	238.38 ±17.46^{b}	276.40 ±21.48^{b}	251.99 ±24.36	254.29 ±17.54^{b}	249.42 ±14.59	238.57 ±19.71	174.76 ±12.29^{a}	195.45 ±13.65^{a}	210.99 ±21.79	0.003*	0.006*	0.424
Protein (g)	79.21 ±6.36^{b}	72.29 ±6.02	69.01 ±4.87	64.94 ±5.97^{ab}	70.53 ±5.14	68.63 ±5.55	57.25 ±4.63^{a}	65.49 ±5.56	69.90 ±6.36	0.033*	0.693	0.871
Fat (g)	50.22 ±6.26	43.34 ±4.16	45.66 ±4.56	47.53 ±16.05	46.87 ±6.28	49.59 ±8.20	46.67 ±5.85	53.49 ±6.60	45.37 ±5.45	0.888	0.470	0.987
Dietary fiber (g)	28.54 ±3.04	32.69 ±2.70	36.10 ±2.54^{b}	28.20 ±20.61	29.90 ±2.38	27.97 ±2.51^{a}	24.70 ±2.75	26.71 ±3.13	25.44 ±3.29^{a}	0.578	0.319	0.025*

Values are Mean±S.E.

Values with different letters($^{a-b}$) within the same column differ significantly(p<0.05) through one-way ANOVA followed by Duncan`s multiple range test.

P_1 : 0wk of each group analyzed. P_2: 1wk of each group analyzed. P_3 : 4wk of each group analyzed.
