# Supplementary material for: Intake of MPRO3 over 4 Weeks Reduces Glucose Levels and Improves Gastrointestinal Health and Metabolism
Source: Microorganisms. 2021 Dec 31;10(1):88. doi: 10.3390/microorganisms10010088 (PMC8780283; doi:10.3390/microorganisms10010088)
Supplement: Supplementary file 1 [file microorganisms-10-00088-s001.zip › Table S5.pdf]

Table S5. Hematological blood test at each time point.

	Index	Unit	A (n= 17)					B (n= 18)					C (n= 16)				
			0 wk	1 wk	4 wk	p^1	p^3	0 wk	1 wk	4 wk	p^1	p^2	0 wk	1 wk	4 wk	p^1	p^2
									Mean (±SD)								
	RBC	10^3 / μL	4.15	4.14	4.09	0.694	0.168	4.25	4.25	4.2	0.892	0.043	4.36	4.34	4.32	0.516	0.27
	(±SD)		0.37	0.32	0.29			0.32	0.31	0.35			0.3	0.3	0.29		
	HGB	g / dL	12.58	12.51	12.46	0.431	0.346	12.96	12.84	12.81	0.119	0.638	13.83	13.63	13.65	0.048	0.775
	(±SD)		1.08	0.89	0.74			0.84	0.83	0.86			0.75	0.85	0.83		
	HCT	%	38.13	38.17	36.94	0.88	0.016	39.14	39.26	37.99	0.655	0	41.01	41.07	40.33	0.967	0.052
	(±SD)		3.32	2.6	2.18			2.4	2.47	2.75			2.04	2.22	1.92		
	MCV	fL	91.91	92.29	90.35	0.004	0.002	92.29	92.46	90.67	0.436	0	94.12	94.74	93.43	0.057	0.004
RBC	(±SD)		3.8	3.78	3.28			3.02	2.88	3.22			3.33	3.51	3.2		
	MCH	pg	30.32	30.22	30.49	0.283	0.061	30.54	30.26	30.58	0.004	0.002	31.73	31.42	31.59	0.002	0.021
	(±SD)		1.27	1.32	1.3			1.2	1.02	1.24			1.12	0.98	1.08		
	MCHC	g / dL	33	32.76	33.76	0.047	0.001	33.09	32.71	33.74	0.006	0	33.73	33.17	33.84	0.001	0.007
	(±SD)		0.76	0.59	0.7			0.57	0.46	0.92			0.9	0.76	0.89		
	RDW-SD	fL	42.46	43.16	41.41	0.001	0.025	43.07	43.21	41.8	0.548	0	43.04	43.86	42.81	0.029	0.03
	(±SD)		2.98	2.86	2.62			2.03	1.63	1.79			2.85	2.9	2.67		
	RDW-CV	%	12.68	12.85	12.58	0.009	0.306	12.83	12.84	12.66	0.805	0.011	12.51	12.68	12.61	0.003	0.189
	(±SD)		0.59	0.49	0.5			0.54	0.55	0.43			0.48	0.42	0.44		
	WBC		6.26	6.8	5.96	0.076	0.154	6.16	5.94	5.67	0.31	0.143	5.27	5.46	5.74	0.172	0.678
	(±SD)		1.39	1.24	1.35			0.78	1.08	1.22			1.41	1.49	1.48		
	NEUT		3.46	3.88	3.31	0.164	0.405	3.33	3.08	3.08	0.096	0.987	3.69	3.5	3.2	0.345	0.681
	(±SD)		1.14	1.37	1.09			0.7	0.84	1			1.42	1.45	1.17		
	NEUT(%)		54.45	55.7	55.11	0.509	0.692	53.81	51.61	53.96	0.061	0.141	59.14	56.18	54.81	0.56	0.408
	(±SD)		8	11.79	9.36			8.05	9.24	9.57			9.41	8.8	10.14		
	LYMPHO		2.33	2.47	2.19	0.059	0.211	2.36	2.42	2.12	0.576	0.002	1.98	2.16	2.1	0.06	0.44
	(±SD)		0.58	0.63	0.67			0.51	0.64	0.56			0.59	0.62	0.65		
	LYMPHO(%)		37.94	37.61	37.13	0.846	0.628	38.45	40.86	37.7	0.021	0.067	33.13	36.83	37.35	0.141	0.107
	(±SD)		7.87	11.08	9.31			8.11	9.32	8.38			7.81	7.77	9.25		
WBC	MONO	10^3 / μL	0.32	0.31	0.33	0.729	0.488	0.31	0.26	0.31	0.026	0.128	0.32	0.27	0.3	0.047	0.007
	(±SD)		0.1	0.12	0.1			0.09	0.08	0.11			0.11	0.08	0.1		
	MONO(%)		5.09	4.56	5.58	0.125	0.08	4.98	4.33	5.43	0.102	0.037	5.51	4.53	5.38	0.013	0.011
	(±SD)		1.24	1.49	1.3			1.38	1.19	1.81			2.23	1.25	1.83		
	EO		0.13	0.13	0.11	0.681	0.021	0.14	0.16	0.13	0.135	0.054	0.12	0.13	0.12	0.912	0.965
	(±SD)		0.07	0.07	0.07			0.13	0.14	0.12			0.1	0.11	0.09		
	EO(%)		2.12	1.81	1.82	0.198	0.127	2.31	2.71	2.44	0.057	0.205	1.92	2.16	2.14	0.632	0.83
	(±SD)		1.17	0.94	1.12			2.22	2.54	2.37			1.49	1.5	1.6		
	BASO		0.03	0.02	0.02	0.088	0.186	0.03	0.03	0.03	0.547	0.111	0.02	0.02	0.02	0.774	0.774
	(±SD)		0.02	0.01	0.01			0.02	0.02	0.02			0.01	0.01	0.01		
	BASO(%)		0.4	0.32	0.36	0.079	0.519	0.44	0.49	0.47	0.166	0.462	0.3	0.31	0.31	0.451	0.887
	(±SD)		0.24	0.21	0.24			0.22	0.24	0.26			0.14	0.17	0.19		
	PLT	10^3 / μL	232.65	246.18	217.53	0.001	0.006	240.17	253.17	231.61	0.009	0	232.25	240.44	227.75	0.005	0.001
	(±SD)		52.53	54.76	54.4			46.24	52.64	52.71			45.19	39.58	35.03		
	PDW	fL	12.06	12.28	11.45	0.404	0.017	12.52	12.94	12.13	0.009	0	10.85	11.81	10.93	0.135	0.021
	(±SD)		1.93	1.59	1.74			1.27	1.21	1.31			1.03	0.98	0.98		
	MPV	fL	10.48	10.51	10.23	0.616	0.009	10.74	10.88	10.56	0.114	0.001	9.96	10.26	9.89	0.232	0.134
PLT	(±SD)		0.92	0.85	0.89			0.62	0.58	0.61			0.49	0.44	0.42		
	PLCR	%	28.45	28.56	26.18	0.873	0.009	30.58	32.03	29.2	0.034	0.001	24.08	26.53	23.68	0.167	0.081
	(±SD)		7.88	7.26	7.32			5.22	4.98	5.41			4.37	3.66	3.6		
	PCT	%	0.24	0.26	0.22	0.004	0	0.26	0.28	0.24	0.007	0	0.23	0.25	0.22	0.005	0.001
	(±SD)		0.05	0.05	0.05			0.05	0.05	0.05			0.04	0.04	0.03		

Abbreviation: WBC, white blood cells; RBC. red blood cells; HGB, hemoglobin; HCT, hematocrit; MCV, mean corpuscular volume; MCH, mean corpuscular hemoglobin; MCHC, mean corpuscular hemoglobin concentration; PLT, platelet; RDW-SD, red cell distribution width; RDW-CV, red cell distribution width - coefficient of variation; PDW, Platelet distribution width; MPV, mean platelet volume; P-LCR, Platelet-large cell ratio; PCT, Procalcitonin.

For statistics, paired t-test was performed.

P_1 : 0wk of each group analyzed. P_2: 1wk of each group analyzed. P_3: 4wk of each group analyzed.
