# Supplementary material for: Intake of MPRO3 over 4 Weeks Reduces Glucose Levels and Improves Gastrointestinal Health and Metabolism
Source: Microorganisms. 2021 Dec 31;10(1):88. doi: 10.3390/microorganisms10010088 (PMC8780283; doi:10.3390/microorganisms10010088)
Supplement: Supplementary file 1 [file microorganisms-10-00088-s001.zip › Table S6.pdf]

Table S6. Chemical blood test at each time point.

	Index	Unit	A (n= 17)					B (n= 18)					C (n= 16)				
			0 wk	1 wk	4 wk	p[1]	p[3]	0 wk	1 wk	4 wk	p[1]	p[2]	0 wk	1 wk	4 wk	p[1]	p[2]
			Mean (±SD)														
Lipid panel	GLU	mg/dL	106.21	89.21	91	0.836	0.038	91.56	91.72	102.78	0.979	0.068	117.94	116.38	112.31	0.323	0.664
	(±SD)		21.7	12.77	17.45			21.4	19.79	21.09			33.9	116.38	42.61		
	HbA1c	%	5.99	6.02	5.86	0.206	0.005	5.8	5.8	5.6	0.331	0	5.8	5.8	5.6	0.697	0
	(±SD)		1.01	1.07	1.14			0.32	0.3	0.37			0.5	0.54	0.45		
	TG	mg/dL	152	131.41	131.76	0.15	0.191	146.94	162.89	163.5	0.413	0.967	149.13	132.44	162.63	0.896	0.75
	(±SD)		85.48	62.9	53.94			75.78	54.53	72.44			61.67	56.68	65.29		
	CHOL	mg/dL	178.18	178.29	170.47	0.978	0.098	200.78	203.11	198.22	0.574	0.171	188.63	190.06	183.25	0.404	0.26
	(±SD)		37.38	36.63	35.05			42.31	39.69	33.4			37.24	39.37	37.43		
	HDL	mg/dL	60.95	60.94	61.09	0.994	0.914	62.32	59.94	60.84	0.019	0.527	54.31	53.08	49.08	0.31	0.906
	(±SD)		10.58	11.16	11.92			10.68	10.6	10.09			12.78	11.37	10.55		
	LDL	mg/dL	88.06	89.92	86.08	0.483	0.472	104.48	108.72	105.47	0.176	0.152	101.39	105.72	99.6	0.049	0.143
	(±SD)		24.33	24.45	24.4			30.59	28.96	25.74			23.77	25.46	22.5		
Renal function	BUN	mg/dL	19.84	16.85	17.48	0	0.008	19.61	17.32	18.98	0	0.053	18.31	17.78	16.63	0.63	0.304
	(±SD)		2.39	3.16	3.92			4.18	3.52	4.49			4.21	3.61	3.62		
	CREA	mg/dL	0.54	0.54	0.57	0.387	0.019	0.55	0.57	0.6	0.105	0.049	0.67	0.72	0.74	0.183	0.029
	(±SD)		0.1	0.09	0.1			0.07	0.09	0.09			0.14	0.15	0.17		
	UA	mg/dL	4.65	4.25	4.59	0.012	0.633	4.73	4.69	4.73	0.749	0.777	5.13	5.24	5.22	0.969	0.261
	(±SD)		1.04	1.12	0.95			0.76	0.69	0.8			1.06	0.99	1.05		
Liver function	AST	U/L	30.76	29.41	29.88	0.341	0.666	28.83	27.22	26.44	0.128	0.194	28.13	28.25	27.31	0.289	0.612
	(±SD)		8.89	7.79	6.49			5.44	4.14	5.05			6.78	7.64	7.7		
	ALT	U/L	22.94	26.47	25.29	0.012	0.161	21.11	20.44	19.61	0.623	0.219	23.88	25	22.44	0.274	0.491
	(±SD)		9.86	12.7	9.16			8.59	7.59	8.86			8.88	9.13	8.6		
	GGT	U/L	25	28.41	26.41	0.3	0.386	17	16.94	16.94	0.938	1	33.94	36.75	34.13	1	0.801
	(±SD)		25.18	37.61	26.57			5.37	5.46	5.59			30.38	30.68	29.12		
	ALP	U/L	73.71	73.59	71.18	0.974	0.285	77.83	79.39	73.39	0.6	0.047	66.06	69.5	63.69	0.361	0.399
	(±SD)		18.47	22.88	16.85			18.06	27.54	20.01			17.71	14.01	19.26		
	TBILC	mg/dL	0.59	0.55	0.6	0.252	0.843	0.55	0.51	0.54	0.431	0.329	0.7	0.62	0.7	0.869	0.403
	(±SD)		0.16	0.14	0.14			0.18	0.14	0.15			0.24	0.2	0.17		
	ALB	g/dL	4.13	4.01	4.18	0	0.332	4.22	4.04	4.11	0	0.131	4.2	4.08	4.11	0.109	0.472
	(±SD)		0.23	0.21	0.27			0.16	0.14	0.17			0.19	0.2	0.25		
	A/G		1.28	1.28	1.32	1	0.28	1.37	1.34	1.35	0.317	0.707	1.36	1.33	1.3	0.609	0.676
	(±SD)		0.19	0.18	0.21			0.13	0.13	0.2			0.19	0.16	0.1		
	B/C		37.53	31.71	30.65	0.001	0	36.22	30.78	31.94	0	0.43	27.81	25.5	23.06	0.914	0.057
	(±SD)		7.46	5.21	5.17			6.75	6.39	6.94			6.52	6.21	5.17		
Inflammation	CRP	mg/dL	0.21	0.49	0.23	0.24	0.808	0.1	0.08	0.12	0.574	0.118	0.08	0.13	0.24	0.48	0.289
	(±SD)		0.42	1.33	0.36			0.09	0.04	0.1			0.05	0.11	0.31		
	LDH	U/L	178.47	165.88	173.12	0.005	0.305	186.17	178.61	181.67	0.089	0.467	160.25	152.31	161.75	0.024	0.001
	(±SD)		31.48	28.34	29.93			33.57	34.59	31.91			25.31	20.21	22.57		
	TP	g/dL	7.42	7.18	7.38	0	0.507	7.29	7.13	7.19	0.033	0.327	7.36	7.17	7.27	0.007	0.409
	(±SD)		0.32	0.39	0.32			0.28	0.37	0.29			0.35	0.4	0.36		
	Zonulin	ng/mL			5.22					5.71					3.78		
	(±SD)				5.07					4.02					3.13		

All analyzes were performed using a paired t-test, and mean and SD values are shown in each column. Abbreviations: GLU, glucose; BUN, blood urea nitrogen; CREA, creatinine; UA. uric acid; TP, total protein; ALB, albumin; ALP, Alkaline phosphatase; AST, aspartate aminotransferase; ALT, Alanine Aminotransferase; T-BILC, total-bilirubin; CHOL. cholesterol; TG, triglyceride; GGT, Gamma-glutamyl transferase; HDL cholesterol, high-density lipoprotein cholesterol; LDH, Lactate dehydrogenase; CRP, C-reactive protein; LDL cholesterol, low-density lipoprotein cholesterol; A/G ratio, albumin/Globulin ratio; B/C ratio, BUN/Creatinine ratio.

For statistics, paired t-test was performed.
